# Bcl-2 expression is altered with ovarian tumor progression: an immunohistochemical evaluation

**DOI:** 10.1186/1757-2215-2-16

**Published:** 2009-10-25

**Authors:** Nicole S Anderson, Leslie Turner, Sandra Livingston, Ren Chen, Santo V Nicosia, Patricia A Kruk

**Affiliations:** 1 Department of Pathology and Cell Biology, University of South Florida, Tampa, FL 33612, USA; 2 Office of Clinical Research, University of South Florida, Tampa, FL 33612, USA; 3 H. Lee Moffitt Cancer Center and Research Institute, Tampa, FL 33612, USA

## Abstract

**Background:**

Ovarian cancer is the most lethal gynecologic malignancy. The ovarian tumor microenvironment is comprised of tumor cells, surrounding stroma, and circulating lymphocytes, an important component of the immune response, in tumors. Previous reports have shown that the anti-apoptotic protein Bcl-2 is overexpressed in many solid neoplasms, including ovarian cancers, and contributes to neoplastic transformation and drug-resistant disease, resulting in poor clinical outcome. Likewise, studies indicate improved clinical outcome with increased presence of lymphocytes. Therefore, we sought to examine Bcl-2 expression in normal, benign, and cancerous ovarian tissues to determine the potential relationship between epithelial and stromal Bcl-2 expression in conjunction with the presence of lymphocytes for epithelial ovarian tumor progression.

**Methods:**

Ovarian tissue sections were classified as normal (n = 2), benign (n = 17) or cancerous (n = 28) and immunohistochemically stained for Bcl-2. Bcl-2 expression was assessed according to cellular localization, extent, and intensity of staining. The number of lymphocyte nests as well as the number of lymphocytes within these nests was counted.

**Results:**

While Bcl-2 staining remained cytoplasmic, both percent and intensity of epithelial and stromal Bcl-2 staining decreased with tumor progression. Further, the number of lymphocyte nests dramatically increased with tumor progression.

**Conclusion:**

The data suggest alterations in Bcl-2 expression and lymphocyte infiltration correlate with epithelial ovarian cancer progression. Consequently, Bcl-2 expression and lymphocyte status may be important for prognostic outcome or useful targets for therapeutic intervention.

## Background

Ovarian cancer (OC) currently ranks 5^th ^in cancer related deaths among women in the United States [1] in spite of advances in treatment. Despite an overall OC survival rate of 45%, the five year survival rate for women diagnosed with OC in its early stages is 94%, however these women only make up 19% of reported OC cases [2]. This poor prognosis is, in part, due to a lack of symptoms at early stages as well as lack of a screening marker available to the general public. The ovarian surface epithelium is generally believed to be the origin for the majority of epithelial ovarian cancer cases [3], though current reports of a fallopian tube origin for ovarian cancer have emerged [4,5]. Consequently, the etiology of ovarian cancer is still poorly understood.

A basement membrane consisting mainly of collagenous connective tissue separates the ovarian surface epithelium (OSE), a modified mesothelium, from underlying ovarian stromal tissue [6]. The OSE and stroma both synthesize and secrete components that contribute to deposition of the basement membrane during postovulatory repair [7]. Normal ovarian stroma also produces an array of growth factors, including, but not limited to transforming growth factor-β_1 _(TGF-β_1_) and the hepatocyte growth factor (HGF) receptor c-Met that stimulate autocrine and paracrine-mediated proliferation of the superjacent epithelium. These growth factors tend to be overexpressed in many carcinomas, hence facilitating neoplastic growth [8]. Additionally, stromal-epithelial interactions have been studied in cancers of the bladder, breast, cervix, colon, prostate, and ovary [9-14] and have shown that stromal cells influence epithelial cell growth as well as tumorigenesis.

In addition to the role of the tumor microenvironment, alterations in apoptotic regulation promoting an anti-apoptotic phenotype also support tumor progression. Specifically, Bcl-2, recognized as the prototypical anti-apoptotic protein, is overexpressed in a number of solid tumors, including ovarian cancer, and contributes to neoplastic transformation through inhibition of apoptosis [15], thereby promoting tumor survival.

In contrast, ovarian tumors can also elicit a marked host immune response resulting in the influx of tumor infiltrating lymphocytes into the tumor which recognize antigens expressed on ovarian tumors [16]. The presence of tumor infiltrating lymphocytes in ovarian cancer patients appears to confer a survival advantage [17-19]; however, this immune response is not normally sufficient to inhibit tumor growth over extended periods of time.

While several studies have previously examined Bcl-2 or the contribution of tumor infiltrating lymphocytes separately for ovarian cancer prognosis, we sought to further determine the combined clinical relationship between Bcl-2 expression and lymphocyte filtration for ovarian cancer progression. To our knowledge there have not been any other similar studies to date. Therefore, given the close proximity of tumor cells, their surrounding stroma, and infiltrating lymphocytes, we analyzed the immunohistochemical expression and histological localization of Bcl-2 in ovarian the stromal and epithelial components of normal, benign, and cancer clinical specimens as well as evaluated changes in lymphocyte populations with ovarian tumor progression.

## Methods

### Tissue Specimens

With institutional approval, a previously existing tissue bank was utilized to retrieve a cohort of de-identified women who had undergone primary surgery with complete surgical staging for epithelial ovarian cancer or borderline tumors at the H. Lee Moffitt Cancer Center between 2000 and 2001. This gynecologic oncology procedure database was also used to select women who had undergone oophrectomy due to cystadenoma or had their ovaries removed for unrelated pathology between 2000 and 2001. All tissue specimens were fixed with 10% formalin and paraffin-embedded. Four micron sections were stained with haematoxylin and eosin (H & E) and the slides were reviewed by a pathologist (SVN) to confirm histologic diagnosis according to the International Federation of Gynecology and Obstetrics (FIGO) classification system. The de-identified medical records of these women were reviewed, and tumor pathology was correlated to the immunohistochemical findings. From these observations, the selected ovarian sections were given the following classifications: 2 normal, 17 benign cysts or cystadenomas, and 28 serous papillary carcinomas, though areas of normal ovarian surface epithelium were also present in 5 benign and 4 carcinoma sections.

### Immunohistochemistry

For immunohistochemical studies, further formalin-fixed paraffin sections were cut at 3 microns and dried overnight at room temperature then deparaffinized and rehydrated. Sections were soaked in hydrogen peroxide to block endogenous peroxidase activity. Microwave antigen retrieval was achieved by placing slides in 1× solution of AR-10 (BioGenex #HK057-5K, San Ramon, CA), boiling, and then microwaving for an additional 10 minutes. The specimens were then immunostained on the Dako Autostainer (Dako North America, Inc., Carpinteria, CA) using Monoclonal Mouse Anti-Human Bcl-2 (Clone 124, Dako, Carpinteria, CA) primary antibody (1:40) for 30 minutes and the Dako's EnVision™ + HRP Mouse (DAB+) kit according to the manufacturer's instructions, then counterstained with modified Mayer's haematoxylin, dehydrated through graded alcohol, cleared with xylene, and mounted with resinous mounting medium. In an effort to control variability, all samples were stained at the same time and with the same lot of reagents. Normal tonsil was used as an internal positive control while negative controls were obtained by substitution of primary antibody with normal mouse serum.

### Staining Analysis

Immunohistochemical staining was evaluated independently by four authors (LP, NSA, PAK, and SVN). The pattern of Bcl-2 staining was evaluated as nuclear or cytoplasmic. Amount of stromal and epithelial staining was assessed as percent staining from each section and scored as having either ≤ 50% or >50% positive cells. Staining intensity was also evaluated and classified as negative, weak, moderate, or intense staining. The presence of lymphocyte nests in each section was also observed and counted by observing assemblages of ten or more lymphocytes in 10 random viewings at a total magnification of 100×. The number of lymphocytes in each observed nest was also counted and grouped into 5 categories: <25, 25-50, 50-75, 75-100, or >100 lymphocytes per nest.

### Statistical Methods

SAS version 9.2 (SAS Institute, Cary, NC) was used for statistical analysis of Bcl-2 staining in normal, benign, and cancerous tissue samples. Fisher's exact test was used to test for associations in extent of epithelial and stromal staining between tumor types and staining intensity of epithelial and stromal staining between tissue types. The Cochran-Mantel-Haenszel test was used to test for independence between tissue type and lymphocyte nest size. The generalized linear model, along with pair-wise comparison among tissue types was used to test for differences in tissue type and the number of lymphocyte nests present.

## Results

Immunohistochemical staining was performed on a total of 47 ovarian tissue sections as characterized in table 1. The mean age of the sample population was 62 years (range, 33-88 years) and no significant differences were noted in age among categories. The predominant histologic type for malignant tissue samples was serous (26/28), and patients typically presented with high grade tumors (Grade 3, 19/28). The majority of the malignant samples were also from patients with stage III ovarian cancer (25/28). Samples classified as "other" included follicular cysts and a cystadenofibroma.

**Table 1:** Characteristics of the study cohort.

	**Normal**	**Cysts**	**Cystadenomas**	**WD (Grade 1)**	**MD (Grade 2)**	**PD (Grade 3)**
	**(n = 2)**	**(n = 4)**	**(n = 13)**	**(n = 3)**	**(n = 6)**	**(n = 19)**
**Age (years)**						
Mean (SD)	57.5 (12.0)	58.0 (16.1)	65.0 (10.1)	48.7 (6.5)	64.8 (16.7)	62.7 (14.5)
Range	49-66	48-81	49-79	42-55	33-76	33-88
						
**Histology**						
Serous	N/A	1	11	1	6	19
Mucinous	N/A	0	1	0	0	0
Endometrioid	N/A	0	0	2	0	0
Other	N/A	3	1	0	0	0
						
**Stage**						
I	N/A	N/A	N/A	0	0	1
II	N/A	N/A	N/A	1	0	0
III	N/A	N/A	N/A	2	6	17
IV	N/A	N/A	N/A	0	0	1

N/A indicates category was not applicable to those samples.

WD = well differentiated carcinoma, MD = moderately differentiated serous papillary carcinoma, PD = poorly differentiated serous papillary carcinoma

**Figure 1:**
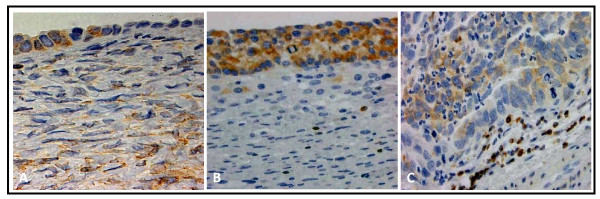
**Bcl-2 staining is most extensive in cysts**. Representative positive Bcl-2 staining in epithelial cells of normal ovary (A), follicular cyst (B), and moderately differentiated serous papillary carcinoma (C). (Original magnification: 200×)

**Figure 2:**
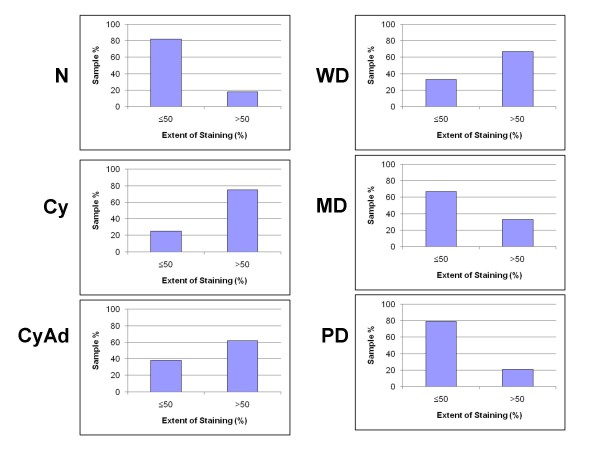
**Extent of epithelial Bcl-2 staining decreases with tumor progression**. Extent of epithelial Bcl-2 staining was observed in each section of normal (N), cyst (Cy), cystadenoma (CyAd), well-differentiated carcinoma (WD), moderately-differentiated serous papillary carcinoma (MD), and poorly-differentiated serous papillary carcinoma (PD) ovarian tissue and categorized as either ≤ 50% or >50% positive staining. Scored sections were graphed as a percent according to total sections of each tumor type.

**Figure 3:**
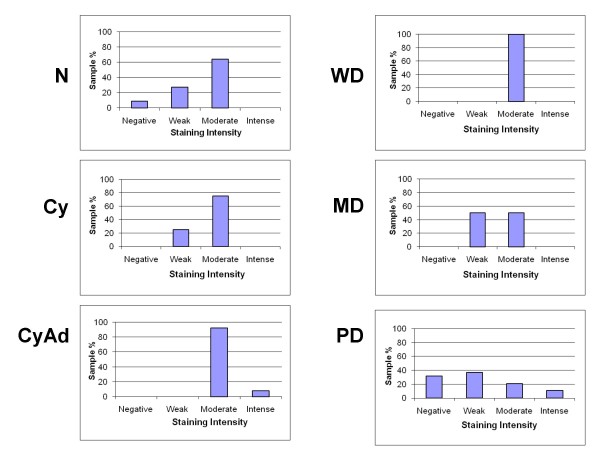
**Intensity of epithelial Bcl-2 staining decreases with tumor progression**. Intensity of epithelial Bcl-2 staining was observed wholly in each section of normal (N), cyst (Cy), cystadenoma (CyAd), well-differentiated carcinoma (WD), moderately-differentiated serous papillary carcinoma (MD), and poorly-differentiated serous papillary carcinoma (PD) ovarian tissue and categorized as having negative, weak, moderate, or intense staining. Scored sections were then graphed as a percent according to total sections of each tumor type.

All tissues in this study, with the exception of four poorly differentiated serous papillary carcinomas, displayed some degree of epithelial and/or stromal Bcl-2 staining. Bcl-2 staining was confined to the cytoplasm in epithelial and stromal cells. While only 2 specimens were classified as normal, there were areas of normal epithelia on 5 benign and 4 cancerous specimens. Due to the small number of normal specimens we were able to procure, we included these additional areas in our normal epithelial analyses. Epithelial Bcl-2 staining was present in 91% (10/11) normal, 100% (17/17) benign, and 79% (22/28) cancer specimens (Figure 1, Table 2). Further, 65% (11/17) benign specimens showed epithelial Bcl-2 staining in more than 50% of their epithelial cells, whereas, 18% (2/11) and 29% (8/28) normal and ovarian cancer tissues, respectively, displayed epithelial Bcl-2 staining to the same extent (Table 2). In the cancerous sections, extent of Bcl-2 epithelial staining tended to decrease with increased tumor grade (Table 2, Figure 2). More than 50% of the epithelial cells stained positive for Bcl-2 in 67% (2/3) of well differentiated carcinomas (WD), 33% (2/6) of moderately differentiated serous papillary carcinomas (MD), and 21% (4/19) of poorly differentiated serous papillary carcinomas (PD) (Table 2, Figure 2). Though there was a trend, these differences were not statistically significant. Similar to the extent of staining, intensity of epithelial Bcl-2 staining was higher in benign samples, with 94% (16/17) of benign sections showing an intensity of moderate or intense, while 64% (7/11) and 43% (12/28) normal and malignant sections, respectively, showed an epithelial staining intensity of moderate or intense degree (Figure 3). More specifically, the epithelial staining intensity in cystadenomas was significantly higher than that in both MD and PD sections (P = 0.02 and P < 0.0001, respectively), but not in WD sections. Comparison among the cancerous samples showed that there was decreased epithelial Bcl-2 intensity with advanced tumor grade (Figure 3); however, these differences did not reach statistical significance.

**Table 2:** Bcl-2 immunoreactivity in ovarian tissue sections.

		**% Positive Epithelium (n)**			**% Positive Stroma (n)**		
			
	**Total**	**Total**	**≤ 50%**	**> 50%**	**Total**	**≤ 50%**	**> 50%**
Normal	2	50 (1)	100 (2)	0	100 (2)	0	100 (2)
Normal within Benign	5	100 (5)	100 (5)	0	**---**	**---**	**---**
Normal within Cancer	4	100 (4)	50 (2)	50 (2)	**---**	**---**	**---**
**Total Normal**	**11**	**91 (10)**	**82 (9)**	**18 (2)**	**100 (2)**	**0**	**100 (2)**
							
Cysts	4	100 (4)	25 (1)	75 (3)	50 (2)	75 (3)	25 (1)
Cystadenomas	13	100 (13)	38 (5)	62 (8)	92 (12)	62 (8)	38 (5)
**Total Benign**	**17**	**100 (17)**	**35 (6)**	**65 (11)**	**82 (14)**	**65 (11)**	**35 (6)**
							
WD	3	100 (3)	33 (1)	67 (2)	100 (3)	66 (2)	33 (1)
MD	6	100 (6)	67 (4)	33 (2)	17 (1)	83 (5)	17 (1)
PD	19	68 (13)	79 (15)	21 (4)	68 (13)	95 (18)	5 (1)
**Total Cancerous**	**28**	**79 (22)**	**71 (20)**	**29 (8)**	**61 (17)**	**89 (25)**	**11 (3)**

WD = well differentiated carcinoma, MD = moderately differentiated serous papillary carcinoma, PD = poorly differentiated serous papillary carcinoma

**Figure 4:**
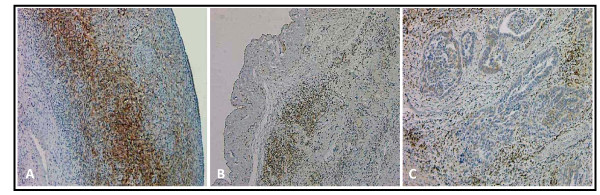
**Stromal Bcl-2 staining decreases with tumor progression**. Representative stromal Bcl-2 staining in normal ovary (A), serous cystadenoma (B), and moderately differentiated serous papillary carcinoma (C). (Original magnification: 40×)

**Figure 5:**
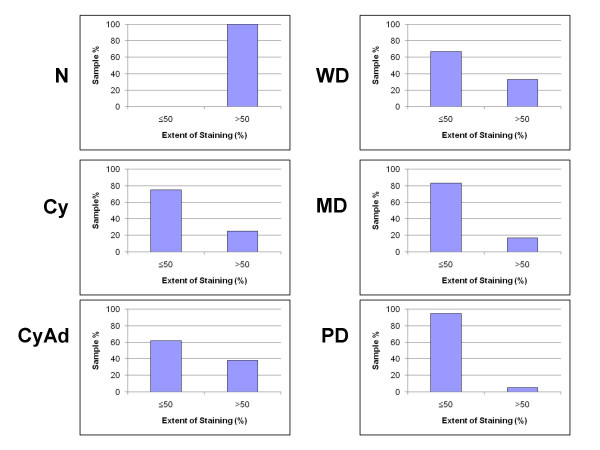
**Extent of stromal Bcl-2 staining decreases with tumor progression**. Extent of stromal Bcl-2 staining was observed in each sections of normal (N), cyst (Cy), cystadenoma (CyAd), well-differentiated carcinoma (WD), moderately-differentiated serous papillary carcinoma (MD), and poorly-differentiated serous papillary carcinoma (PD) ovarian tissue and categorized as either ≤ 50% or >50% positive staining. Scored sections were graphed as a percent according to total sections of each tumor type.

**Figure 6:**
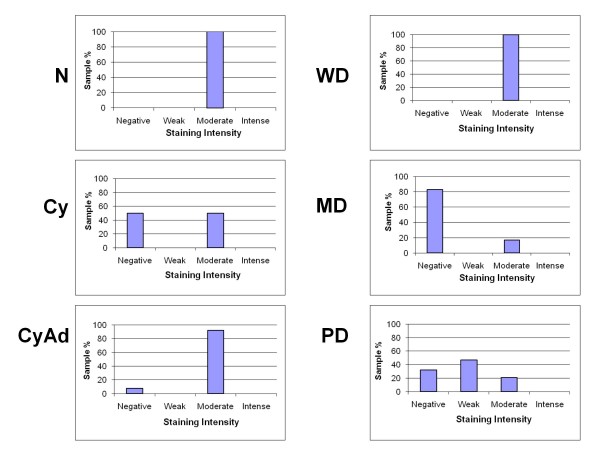
**Intensity of stromal Bcl-2 staining decreases with tumor progression**. Intensity of stromal Bcl-2 staining was observed wholly in each section of normal (N), cyst (Cy), cystadenoma (CyAd), well-differentiated carcinoma (WD), moderately-differentiated serous papillary carcinoma (MD), and poorly-differentiated serous papillary carcinoma (PD) ovarian tissue and categorized as having negative, weak, moderate, or intense staining. Scored sections were then graphed as a percent according to total sections of each tumor type.

Like epithelial staining, stromal Bcl-2 staining also decreased with malignant progression (Figure 4). One hundred percent (2/2) normal, 82% (14/17) benign, and 61% (17/28) malignant samples stained positive for stromal Bcl-2 (Table 2). While 100% (2/2) of the normal specimens were found to have a positive Bcl-2 staining in more than 50% of the stromal cells, 35% (6/17) of benign tumors had Bcl-2 expression in more than 50% of the stromal cells (Figure 5). In contrast, only 11% (3/28) of ovarian cancer sections had more than 50% of their stromal cells expressing Bcl-2 (Figure 5), and similar to extent of epithelial Bcl-2 staining, differences in extent of stromal Bcl-2 staining between tumor types were not statistically significant with the exception of PD samples having significantly less staining than both normal and cystadenoma samples (P = 0.1 and 0.03, respectively). Stromal intensity was moderate in 100% (2/2) of the normal tissues, and in 82% (14/17) benign tumors (Figure 6). However, moderate stromal intensity decreased to 29% (8/28) in ovarian cancer sections (Figure 6). Stromal Bcl-2 intensity in cystadenomas was significantly different versus intensity in MD and PD specimens (P = 0.003 and P < 0.0001, respectively), while stromal Bcl-2 intensity in cysts was not significantly different from any of the specimens. Likewise, stromal intensity decreased with increased tumor grade with 100% (3/3) WD, 17% (1/6) MD, and 21% (4/19) PD displaying moderate stromal intensity for Bcl-2 staining (Figure 6). Additionally, PD stromal intensity was significantly lower than MD (P = 0.05).

**Figure 7:**
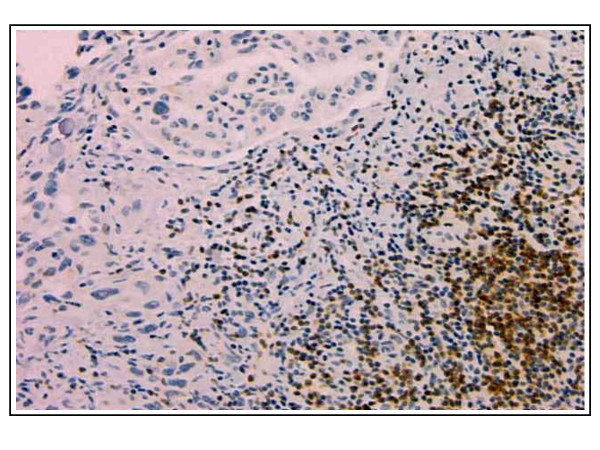
**Lymphocyte nests are more abundant in malignant sections**. Bcl-2 staining of a representative large lymphocyte nest in a poorly differentiated serous papillary carcinoma tumor. (Original magnification: 100×)

In contrast, the average number of lymphocyte nests (defined as aggregates of 10 or more lymphocytes) (Figure 7, Table 3) present per section increased with malignant progression. While normal and benign samples both averaged less than two lymphocyte nests per section, WD sections averaged 3.33 lymphocyte nests per section, and higher grades displayed significantly more lymphocyte nests with MD (P = 0.01) and PD (p = 0.003) sections having averages of 7.67 and 7.84 lymphocyte nests per section, respectively. Due to small sample sizes within tissue subtypes, the samples were divided simply into normal, benign, or cancerous to analyze differences in the sizes of lymphocyte nests (Table 3). Interestingly, the size of lymphocyte nests also significantly increased as tumors became cancerous (p = 0.004). Additionally, lymphocyte population may also be associated with cancer stage because the stage I and II ovarian cancer sections did not contain any lymphocyte nests and, with the exception of one stage III cancer specimen, the stage IV cancer specimen had the highest amount of nests that contained >100 lymphocytes (data not shown).

**Table 3:** Lymphocyte nests and number of lymphocytes in each nest according to tissue type.

**Tumor Type**	**n**	# Lymph Nests*	**# Lymphocytes in Nests**					Average Lymph Nests/Section*
			**<25**	**25-50**	**50-75**	**75-100**	**>100**	
**Normal**	2	0	0	0	0	0	0	0
**Benign**								
**Cyst**	4	6	1	3	2	0	0	1.5
**Cystadenoma**	13	13	8	1	2	1	1	1
**Total Cancerous**	17	19	9	4	4	1	1	1.12
**Cancerous**								
**WD**	3	10	1	4	3	1	1	3.33
**MD**	6	46	11	11	9	2	13	7.67
**PD**	19	149	23	25	23	12	66	7.84
**Total**	28	205	35	40	35	15	80	7.32

*Lymph Nests = aggregates of 10 or more lymphocytes

WD = well differentiated carcinoma, MD = moderately differentiated serous papillary carcinoma, PD = poorly differentiated serous papillary carcinoma

## Discussion

While there have been several studies examining Bcl-2 expression with ovarian tumor progression or the prognostic importance of the presence of lymphocytes for clinical outcome in ovarian cancer, our study is the first to examine Bcl-2 expression in both epithelial and stromal cells as well as lymphocyte distribution with ovarian cancer progression. In agreement with previous studies [20-22] we found that over 50% of ovarian cancers stained for Bcl-2, but we also detected Bcl-2 staining in normal and benign ovarian specimens. Further, epithelial Bcl-2 staining was greater in normal and benign ovarian specimens compared with cancer specimens. This is in agreement with other studies [23-25] which reported greater Bcl-2 expression in normal and benign specimen compared to cancer samples. Chan et al. [23] proposed that decreased Bcl-2 expression with tumor progression resulted from the dysregulation of Bcl-2 normally required to maintain physiological function and integrity of the normal ovarian surface epithelium. Similarly, other studies [20,23,26] have reported an inverse relationship between epithelial Bcl-2 expression and tumor grade. For example, Baekelandt et al. [27] found only 39% of stage III epithelial ovarian carcinomas displayed immunoreactivity to Bcl-2 in more than 5% of the tumor cells. They did not compare these levels to Bcl-2 expression in normal ovarian tissue, but they did conclude that Bcl-2 expression was inversely related to tumor aggressiveness. In the present study, 57% (16/28) of the ovarian cancer specimens showed positive Bcl-2 staining in more than 5% of the tumor cells regardless of cancer stage. However, when only stage III ovarian cancer specimens were considered, 42% (8/19) of the samples demonstrated positive Bcl-2 staining in more than 5% of the tumor cells (data not shown) which is very similar to findings reported by Baekelandt et al [27]. Interestingly, we have recently reported increased levels of urinary Bcl-2 in ovarian cancer patients [28] suggesting that reduced epithelial Bcl-2 staining with tumor progression may reflect a transition from cellular expression of Bcl-2 to secreted Bcl-2 associated with disease progression.

Further, normal ovarian endocrine and reproductive function depends on a multifaceted and dynamic microenvironment that involves coordinated cell-cell interactions [29]. Likewise, stromal-epithelial interactions, as seen in breast carcinomas [30-32], play an important role in determining ovarian malignant progression. This is supported by the observation that ovarian surface epithelium (OSE) tumor cells are closely associated with their surrounding stromal cells [33]. Interestingly, conditioned media from normal stromal cells inhibits proliferation of SKOV3 and Caov3 ovarian cancer cell lines *in vitro *[29], while nude mice co-injected with SKOV3 or OCC1 ovarian cancer cells and normal stromal cells display a slower onset of tumor formation and rate of tumor growth compared to mice injected with cancer cells alone [13]. Additionally, precursors of OSE tumors, such as hyper- and metaplastic changes of the OSE and associated inclusion cysts, are related to stromal hyperplasia [34]. In the present study, we found that stromal Bcl-2 staining decreased with malignant progression and the intensity of stromal Bcl-2 expression was inversely related to tumor grade, possibly suggesting that alterations in stromal components might promote tumor progression. Taken together, these findings support a role of tumor-stromal interactions in the regulation of tumorigenesis as well as tumor progression in epithelial ovarian cancer.

Lastly, OC is a highly immunogenic disease which triggers the influx of a large number of lymphoid cells to the tumor site. Lymphocytes play a major role in the host immune response since stimulated lymphocytes release cytokines, antibodies, and growth factors necessary for immune-mediated tumor cell lysis [35]. Consequently, the presence of T cells is generally associated with an improved clinical outcome in advanced ovarian carcinoma. Adams et al. [36] showed that ovarian cancer patients who have tumors with a high frequency of intraepithelial T cells, specifically CD8^+ ^T cells, have a significantly better 5-year survival rate than patients whose tumors have a low frequency of intraepithelial CD8^+ ^T cells. Likewise, Clarke et al. found that the presence of intraepithelial CD3^+ ^and CD8^+ ^T cells was associated with improved survival in patients with serous ovarian carcinomas, but not patients with endometrioid or clear cell carcinomas [18]. These latter findings may be related to the presence of CD8^+ ^T lymphocytes in underlying tumor stroma correlating with vascular invasion thereby potentiating tumor growth in endometrioid carcinoma [16]. In the present study, we found an increased number of lymphocyte nests with malignant transformation in ovarian specimens and the size of lymphocytes nests also increased significantly with tumor progression; however we did not have any information on patient survival to report any prognostic data. Given that lymphocytes secrete TGF-β [37] which can promote mesenchymal cell growth [38], focal areas of lymphocytes, then, may support growth of higher grade ovarian tumors, especially as that pertains to ovarian epithelial cells that have undergone epithelial to mesenchymal transition characteristic of ovarian cancer progression [39]. TGF-β is also thought to have angiogenic properties [40] which would additionally benefit tumor growth. Our findings of increased lymphoid aggregates present with ovarian cancer progression are in agreement with other cancers including lymphoma [41], breast cancer [42], and melanoma [43]. However, whether these lymphocytes assist in the antitumor response or promote tumor growth remains unclear since the role that they play may very well be disease-specific.

## Conclusion

In this pilot study, it appears that alterations in Bcl-2 expression and the number of lymphocytes may be to be correlated with ovarian cancer progression. Clearly, then, further studies with additional samples are warranted since, the combination of Bcl-2 expression and lymphocyte status may be important for prognostic outcome or provide useful targets for therapeutic intervention in patients with epithelial ovarian cancer.

## Competing interests

The authors declare that they have no competing interests.

## Authors' contributions

PAK, SVN, NSA, and LT reviewed and analyzed immunohistochemistry sections while NSA and LT prepared the figures. NSA contributed to writing of the manuscript. SL performed immunohistochemistry. SVN procured specimens and verified de-identified histologic diagnoses and clinical information. RC performed statistical analyses. PAK developed and oversaw the project from its planning through execution and preparation of this manuscript. All authors read and approved the final version of the manuscript.
